# Supplementary material for: EnzML: multi-label prediction of enzyme classes using InterPro signatures
Source: BMC Bioinformatics. 2012 Apr 25;13:61. doi: 10.1186/1471-2105-13-61 (PMC3483700; doi:10.1186/1471-2105-13-61)
Supplement: Addtional file 5 — The Java code to format the data files, evaluate and predict. The file enzml_java_code.tar.gz contains the Java code used to format database data to ARFF and XML formats, to execute cross and train-test (jackknife) evaluations and to record evaluation results to database. More information is included in the readme.txt file and the Javadoc files. The code can be used with a MySQL database. To use a different database software, other JDBC drivers might be required. [file 1471-2105-13-61-S5.gz › java_code/utils/doc/uk/ac/ed/inf/utils/database/package-frame.html]

uk.ac.ed.inf.utils.database


uk.ac.ed.inf.utils.database

Classes 
  
DbConn
  
DbCreator
  
DbManaged
  
DbManager
  
DbReader
  
DbUtils
  
DbWriter
  
Managed
  
Manager
  
ResultSetUtils
  
SqlUtils
  
Table
  
TableColumn
  
TableCreator
  
TableManaged
  
TableManager
  
TableReader
  
TableRow
  
TableWriter
